# Effective Immunohistochemistry Pathology Microscopy Image Generation Using CycleGAN

**DOI:** 10.3389/fmolb.2020.571180

**Published:** 2020-10-22

**Authors:** Zidui Xu, Xi Li, Xihan Zhu, Luyang Chen, Yonghong He, Yupeng Chen

**Affiliations:** ^1^Department of Life and Health, Tsinghua Shenzhen International School, Shenzhen, China; ^2^Department of Gastroenterology, Peking University Shenzhen Hospital, Shenzhen, China; ^3^School of Traditional Chinese Medicine, Capital Medical University, Beijing, China; ^4^Department of Computer Science, Pennsylvania State University, University Park, PA, United States; ^5^Peng Cheng Laboratory, Shenzhen, China

**Keywords:** immunohistochemistry pathology microscopy image, medical image generation, CycleGAN, conditional GAN, multiple instances learning

## Abstract

Immunohistochemistry detection technology is able to detect more difficult tumors than regular pathology detection technology only with hematoxylin-eosin stained pathology microscopy images, – for example, neuroendocrine tumor detection. However, making immunohistochemistry pathology microscopy images costs much time and money. In this paper, we propose an effective immunohistochemistry pathology microscopic image-generation method that can generate synthetic immunohistochemistry pathology microscopic images from hematoxylin-eosin stained pathology microscopy images without any annotation. CycleGAN is adopted as the basic architecture for the unpaired and unannotated dataset. Moreover, multiple instances learning algorithms and the idea behind conditional GAN are considered to improve performance. To our knowledge, this is the first attempt to generate immunohistochemistry pathology microscopic images, and our method can achieve good performance, which will be very useful for pathologists and patients when applied in clinical practice.

## Introduction

Immunohistochemistry (IHC) detection technology, such as staining with Ki-67 reagent, plays an important role in tumor detection. About 5–10% of patients with tumors cannot be detected accurately only with hematoxylin-eosin (HE) stained pathology microscopic images. Luckily, with the rapid development of IHC detection technology, many difficult tumors can be detected, especially undifferentiated or poorly differentiated tumors. Although IHC detection technology is a more accurate method, making Ki-67 pathology microscopic images costs a large amount of money and time. Considering the surprising performance of deep learning technology in medical image analysis region (Wang et al., 2016; Liu et al., 2017; Li and Ping, 2018), especially generating synthetic medical images using generative adversarial networks (GAN) and its variants (Goodfellow et al., 2014; Hoffman et al., 2017; Nie et al., 2017; Frid-Adar et al., 2018a,b; Han et al., 2018; Kazeminia et al., 2018; Mahmood et al., 2018; Shin et al., 2018), generating synthetic Ki-67 pathology microscopy images from HE pathology microscopy images using GAN would be a good choice.

However, generating synthetic Ki-67 microscopy images from HE pathology microscopy images is a challenging task. The first reason is the unpaired dataset. Because a pathology slice is not usually allowed to be stained twice, in this paper, the HE pathology microscopy image and its corresponding Ki-67 pathology microscopy image are not pixel-aligned. The second reason is the difficulty of achieving professional pathological annotations. Unlike any other common computer vision tasks, the annotations of pathology microscopy images can only be completed and checked by professional pathologists, so it is extremely hard to get a large fully and accurately annotated microscopy image dataset. The third reason is the difficulty of considering class-related information from HE pathology microscopy images to Ki-67 pathology microscopy images. The adversarial training process will align some feature vectors between different domains (Song et al., 2019), but the class-related feature vectors are what we need, and we need to handle them.

In recent years, with the development of deep learning technology, many researchers have tried their best to address these drawbacks. CycleGAN was proposed for unpaired image datasets when generating synthetic images (Zhu et al., 2017). By introducing cycle loss functions during the adversarial training process, the generator finds an accurate mapping between two different domains with unpaired datasets. In this view, CycleGAN is the proper way for unpaired pathology microscopy image datasets. With incomplete or lacking annotations of pathology microscopy images, semi-supervised learning-based methods, unsupervised learning-based methods and self-supervised learning-based methods have been introduced to work on datasets with partial annotations or without any annotation and these methods have proven to be useful (Campanella et al., 2019; Xu G. et al., 2019). Among these methods, multiple instances learning (MIL) algorithms have been applied successfully with unannotated pathology microscopy images, so they have been adopted in this paper (Xu G. et al., 2019). Actually, the adversarial training process aims to align some feature vectors extracting from real images or fake images (Nguyen et al., 2017). When the feature vector that makes the largest contribution to the discriminator are aligned well, then the discriminator would neglect other feature vectors. In our work, we found that when training with small pathology microscopy patches, morphological feature vectors are selected for alignment during adversarial training process. However, what we need is to align class-related feature vectors. The idea behind conditional GAN is extended to address this problem by treating the class label as the additional channel of the input patch, and the class label can be obtained from MIL algorithms (Mirza and Osindero, 2014). By teaching the generator to focus on class-related information, the model will achieve better performance.

**FIGURE 1:**
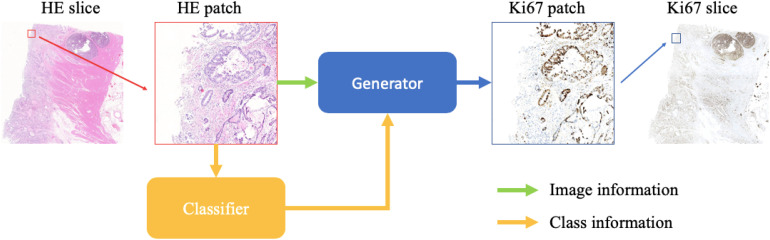
The evaluation process of our method. After patch extraction, the HE pathology patches will be fed into a trained classifier at first. Then the class-related information will be combined with the HE pathology patches, and they are the input of the trained generator, which will generate synthetic Ki-67 pathology patches.

We show the schematic representation of our method during the evaluation step in this paper (Figure 1). When we generate a synthetic Ki-67 pathology microscopy image from a specific HE pathology microscopy image, the HE pathology microscopy patch will be fed into a classifier to get its class label. Then this class-related information will be combined with the HE pathology microscopy patch, and they are the input of the generator which will produce a synthetic Ki-67 pathology microscopy patch.

In this paper, the major contributions are in three phases: first, we apply CycleGAN to generate synthetic Ki-67 pathology microscopy images from unpaired HE pathology microscopy images. By introducing the cycle loss function and adjusting the architecture of the networks, CycleGAN is able to find an accurate mapping from the HE domain to the Ki-67 domain. Second, we apply a MIL algorithm to train two classifiers from unannotated HE and Ki-67 pathology microscopy images separately. Both classifiers are used to distinguish tumor patches from normal patches. Last, we apply the idea behind conditional GAN. By treating class-related information as the additional channel of input patches, class-related feature vectors are forced to be aligned well and this strategy is essential to our performance.

The remaining sections are organized as following: section “Materials and Methods” introduces the dataset and our method in detail. In section “Results,” we conduct experiments and show our experimental results. Finally, we conclude and discuss our work in section “Discussion.”

## Materials and Methods

### Dataset

In this paper, we conduct experiments on a neuroendocrine tumor dataset. Formalin-fixed paraffin-embedded tumor samples from 10 patients with neuroendocrine tumors in the Peking University Shenzhen Hospital, China, are used in this work. The samples are stored in the archives of Department of Pathology in Peking University Shenzhen Hospital, and the head of the Department of Pathology approved the usage of the samples in this work. The samples are anonymized. All patient-related data and unique identifiers are removed. These procedures were performed under the supervision and approval of the Ethics Committee in Peking University Shenzhen Hospital.

From each formalin-fixed paraffin-embedded block, we cut two consecutive sections: one for staining with HE and the other for staining with the anti-Ki-67 antibody. During the HE staining process, we used undiluted Mayer’s hematoxylin and 0.5% eosin. During the IHC staining process, we used anti-Ki-67 antibody (Roche United States).

**FIGURE 2:**
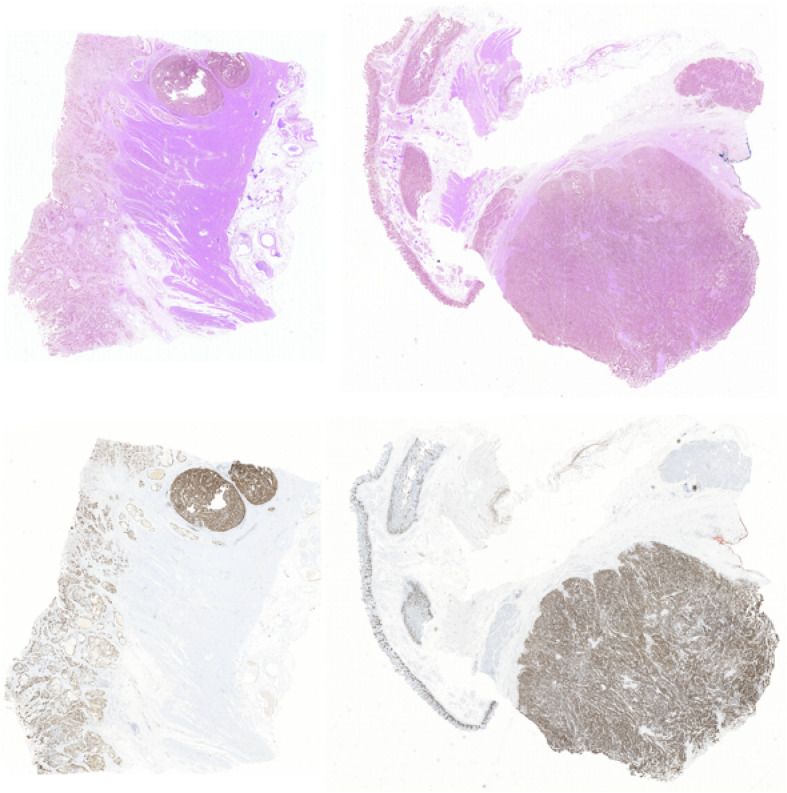
A representation of two HE pathology microscopy images (top) and their corresponding Ki-67 pathology microscopy images (bottom) in our dataset.

In this way, we got 10 HE pathology microscopy images and 10 corresponding Ki-67 pathology microscopy images of neuroendocrine tumors. In all, 7 HE pathology microscopy images and 7 corresponding Ki-67 pathology microscopy images are used as training data, and the rest are used as evaluating data. In this paper, we list the representation of 2 HE pathology microscopy images and their corresponding Ki-67 pathology microscopy images (Figure 2).

### Preprocess

During the MIL training period, for each domain (HE or Ki-67), we want to train a binary classifier to classify tumor patches or normal patches. However, our data are unannotated. So two preprocess steps are necessary. The first step is foreground extraction. There exist three kinds of patches from HE pathology microscopy images or Ki-67 pathology microscopy images: tumor patches including at least one tumor cell, normal patches including only normal cells, and background patches including only background. But the MIL algorithm is used to train a binary classifier to classify two kinds of patches, so the background patches should be removed. In this paper, an OTSU algorithm (Xu G. et al., 2019) is used to extract foreground from HE pathology microscopy images and Ki-67 pathology microscopy images. The second step is extracting large patches with weak annotations. In this paper, HE pathology slices and Ki-67 pathology slices are all positive slices containing tumor cells. But the MIL algorithm works with positive samples and negative samples with weak annotations. For this reason, the foreground of HE pathology microscopy images and Ki-67 pathology microscopy images is cropped into large patches (2,240 × 2,240 pixels), and then we can label these large patches manually. With this preprocess, we can get two weakly annotated datasets (one HE large patch dataset and one Ki-67 large patch dataset), and they can be used to train two classifiers (one is used in the HE domain, and the other one is used in the Ki-67 domain) using the MIL algorithm.

During the CycleGAN training period, the background of pathology microscopy images should not be removed because the generator is required to generate a synthetic background at the same time. The extraction of large patches is also removed. The pathology microscopy images should be cropped into small patches (224 × 224 pixels) directly.

### Method

**FIGURE 3:**
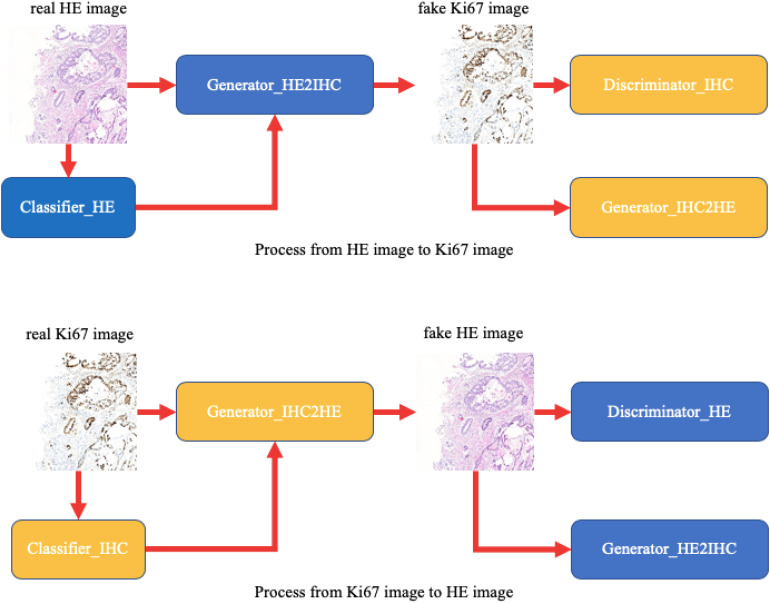
A schematic representation of our method. Unlike raw CycleGAN, during the adversarial training process, the classifiers will extract class-related information, which will be introduced into the training process. This strategy will force the class-related feature vectors to be aligned and then improve our performance.

In this paper, we provide a schematic representation of our method (Figure 3). At first, we train two binary classifiers classifying tumor patches or normal patches with unannotated HE and Ki-67 pathology microscopy images using the MIL algorithm. During the CycleGAN training period, the input patches will be fed into the above classifiers in order first to get its class-related information. Then, following what conditional GAN does, class-related information will be considered as the additional channel of the input patch. This strategy will force class-related feature vectors to be aligned accurately during the adversarial training process. Note that our method is working with an unannotated and unpaired dataset, and thus it can be applied to many other tasks.

### MIL for Getting Class-Related Information

There have been many methods based on deep learning technology for tumor cell detection in pathology microscopy images. A dataset with annotations from professional pathologists is essential for the models’ feasibility and performance, but it is too hard to get such a dataset. To address this drawback, in this paper, we apply the MIL algorithm to collect class-related information from an unannotated dataset.

During the MIL training period, the HE and Ki-67 pathology microscopy images are cropped into large patches (2,240 × 2,240 pixels), and these large patches are manually labeled as tumor patches or normal patches. A large patch is assigned a tumor label if it contains at least one tumor cell, while it is assigned a normal label if it contains only normal cells. After we have labeled all large patches, small patch extraction is necessary for low GPU memory, and each small patch is set at 224 × 224 pixels. Each iteration consists of an evaluation step and a training step. During the evaluation step, for each large patch, a classifier evaluates all small patches from this large patch and then one selected small patch is labeled. The small patch with the largest predictive probability is the one selected, and it should be considered the representative small patch for the large patch. For a large tumor patch, the selected small patch is assigned the tumor label. For large normal patch, the selected small patch is assigned the normal label. After the evaluation step, a training dataset of selected small patches with labels can be used to train the classifier. The trained classifier would evaluate all small patches in the next iteration. Finally, we will achieve two classifiers for classifying tumor patches or normal patches from HE and Ki-67 pathology microscopy images. And they can be used to get class-related information from unannotated data.

During the MIL training process, the number of training samples is listed in Table 1. What’s more, the classifiers used are ResNet34 (He et al., 2016).

**TABLE 1:** MIL and CycleGAN dataset.

	**Training dataset**		**Evaluation dataset**	
	**HE**	**Ki-67**	**HE**	**Ki-67**
MIL	307/87	359/78	35/35	46/40
CycleGAN	34121	33293	4396	4031

*The table above shows the number of samples used during the MIL training process. During the MIL training process, they are the number of positive samples/the number of negative samples. The training data are at the large patch level (2,240 × 2,240 pixels) during the MIL training process and at small patch level (2,24 × 2,24 pixels) during the CycleGAN training process.*

### CycleGAN for an Unpaired Dataset

**FIGURE 4:**
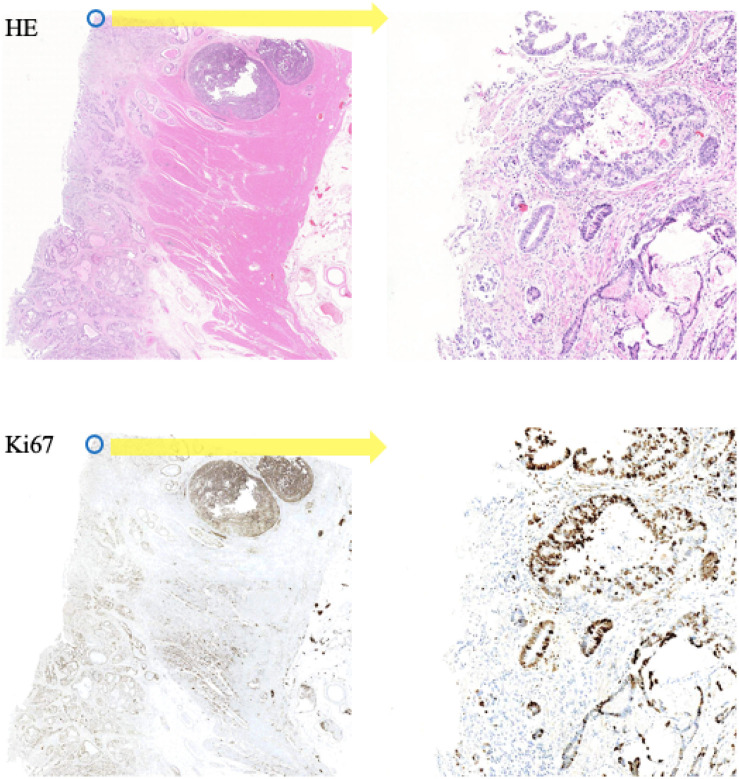
A representation of our dataset. Because the Ki-67 pathology slice is the consecutive slice near HE pathology slice, these two pathology microscopy images are almost same at image level, but they are different at pixel level or patch level.

In this paper, the corresponding Ki-67 pathology slice is the consecutive slice near the HE pathology slice. This means they are same mostly at the image level, but at the pixel level, they are different and unpaired, just as shown in Figure 4. As a result, CycleGAN is an appropriate solution for our task.

During the CycleGAN training period, HE and Ki-67 pathology microscopy images are directly cropped into small patches (224 × 224 pixels), including background. In this paper, the generator in raw CycleGAN has been replaced by a more complicated generator because we found that in raw CycleGAN, the discriminator would learn much faster than the generator when training with our data, so the generator could not learn anything and fails to generate synthetic Ki-67 pathology microscopy images of high quality.

During CycleGAN training process, the number of training samples is listed in Table 1 and the detailed network configuration is shown in Table 2.

**TABLE 2:** CycleGAN configuration.

**Generator**	**Discriminator**
**Conv (in_c = 3, out_c = 64, k = 7, s = 1, *p* = 3)**	**Conv(in_c = 3, out_c = 64, k = 7, s = 1, *p* = 3)**
InstanceNorm(64)	LeakyReLU(0.2)
ReLU()	Conv(in_c = 64, out_c = 128, *k* = 3, *s* = 2, *p* = 1)
Conv(in_c = 64, out_c = 128, *k* = 3, *s* = 2, *p* = 1)	InstanceNorm(128)
InstanceNorm(128)	LeakyReLU(0.2)
ReLU()	Conv(in_c = 128, out_c = 256, k = 3, s = 2, p = 1)
Conv(in_c = 128,out_c = 256, *k* = 3, *s* = 2, *p* = 1)	InstanceNorm(256)
InstanceNorm(256)	LeakyReLU(0.2)
ReLU()	Conv(in_c = 256,out_c = 512, *k* = 3, *s* = 2, *p* = 1)
Conv(in_c = 256, out_c = 256, *k* = 3, s = 1, *p* = 1)	InstanceNorm(512)
InstanceNorm(256)	LeakyReLU(0.2)
ReLU()	Conv(in_c = 512,out_c = 1, *k* = 4, *s* = 1, *p* = 1)
Conv(in_c = 256,out_c = 256, *k* = 3, *s* = 1, *p* = 1)	AveragePool()
InstanceNorm(256)	
Conv_transpose(in_c = 256, out_c = 128, *k* = 3, *s* = 2, p = 1, p_o = 1)	
InstanceNorm(128)	
ReLU()	
Conv_transpose(in_c = 128, out_c = 64, *k* = 3, *s* = 2, p = 1, p_o = 1)	
InstanceNorm(64)	
ReLU()	
Conv(in_c = 64, out_c = 3, *k* = 7, *s* = 1, *p* = 3)	
Tanh()	

*The table above shows the detailed configuration of generator and discriminator in CycleGAN framework during CycleGAN training process. Specifically, Conv is convolution, InstanceNorm is instance normalization, ReLU is ReLU activation function, Conv_transpose is transposed convolution, Tanh is Tanh activation function, AveragePool is the average pooling function, in_c is input channel, out_c is output channel, k is kernel size, s is stride size, p is padding size, and p_o is output padding size.*

### Conditional GAN for Class-Related Alignment

In the generation of synthetic images, the adversarial training process of GAN and its variants can be treated as learning an accurate mapping of some feature vectors between different domains. From the viewpoint of domain adaptation, the discriminator in GAN focuses on some feature vectors while neglecting other feature vectors that are less important. The situation is the same with CycleGAN. In our work, we found that raw CycleGAN would neglect class-related feature vectors during the adversarial training process. However, we need to consider the class-related information because Ki-67 pathology microscopy images are used to detect tumor cells. In order to align class-related feature vectors, the idea behinds conditional GAN is introduced to take class-related information into consideration (Xu Z. et al., 2019). In detail, the input patch of our CycleGAN is not only the HE microscopy patch or the Ki-67 microscopy patch, but also their class-related information generated from the classifier training with the MIL algorithm.

**FIGURE 5:**
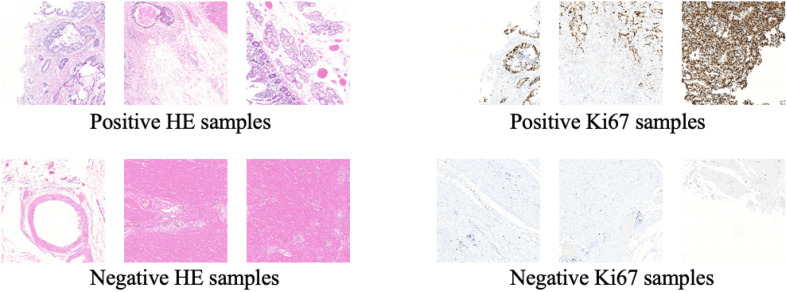
Some training samples during MIL training process.

**FIGURE 6:**
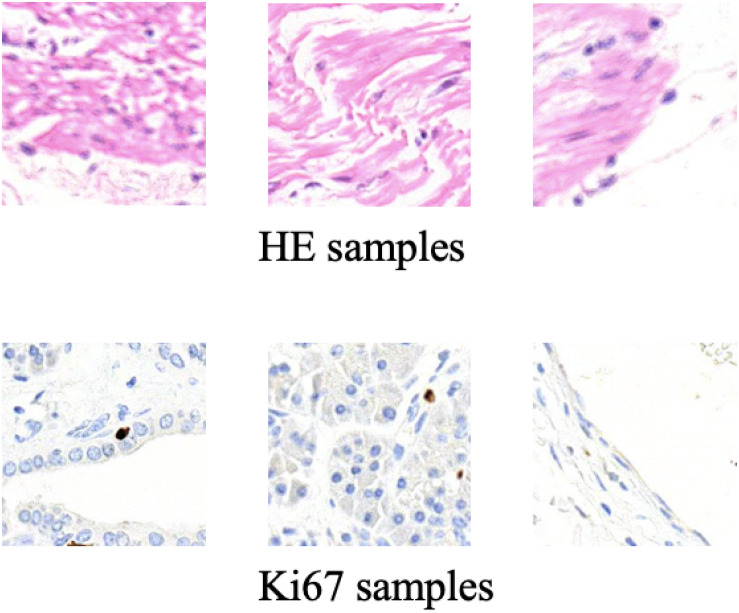
Some training samples during CycleGAN training process.

Some training samples during MIL training process and CycleGAN training process will be listed to better understand the differences between them. Figure 5 shows some training samples during the MIL training process while Figure 6 shows some training samples during the CycleGAN training process.

### Training Configuration

Our method is implemented in the PyTorch framework on an Ubuntu platform. All experiments are conducted on a computer equipped with an NVIDIA GTX 2080 Ti graphic card with 11 GB of memory. During the training stage of our method, the deep neural networks are trained with Adam stochastic gradient descent algorithm. We use the learning rate of 0.0001 for 100 training epochs during the MIL training process and CycleGAN training process, and we will save the models each epoch. Among these 100 saved models, the one achieving the best result on the validation set is selected as the final model.

As for the loss function, cross entropy loss is used to train two classifiers during the MIL training process. During CycleGAN training process, just as with normal CycleGAN, the loss function for the discriminator is binary cross-entropy loss, and the loss function for the generator is mean square error loss.

## Results

### Metrics

Unlike usual image generation tasks, in this paper, the evaluation metric is lacking because a pathology slice is not stained twice usually. It means that there is no way to get the pixel-aligned Ki-67 pathology microscopy image from a specific HE pathology microscopy image. To address this question, we have got the consecutive Ki-67 pathology slice near HE pathology slice. In this paradigm, the HE pathology microscopy image and its corresponding Ki-67 pathology microscopy image appear similar at image level. But they are different at the patch level. Considering this phenomenon, we evaluate our proposed method by image-level visualization and patch-level visualization. The image-level visualization can be used to evaluate the alignment of global feature vectors, and the patch-level visualization can be used to evaluate the alignment of class-related feature vectors. Moreover, we calculate the ratio of positive cells to all cells in a real Ki-67 pathology microscopy image, and its corresponding fake Ki-67 pathology microscopy image. These two ratios should be as close as possible.

### Visualization of Patch Classification

**FIGURE 7:**
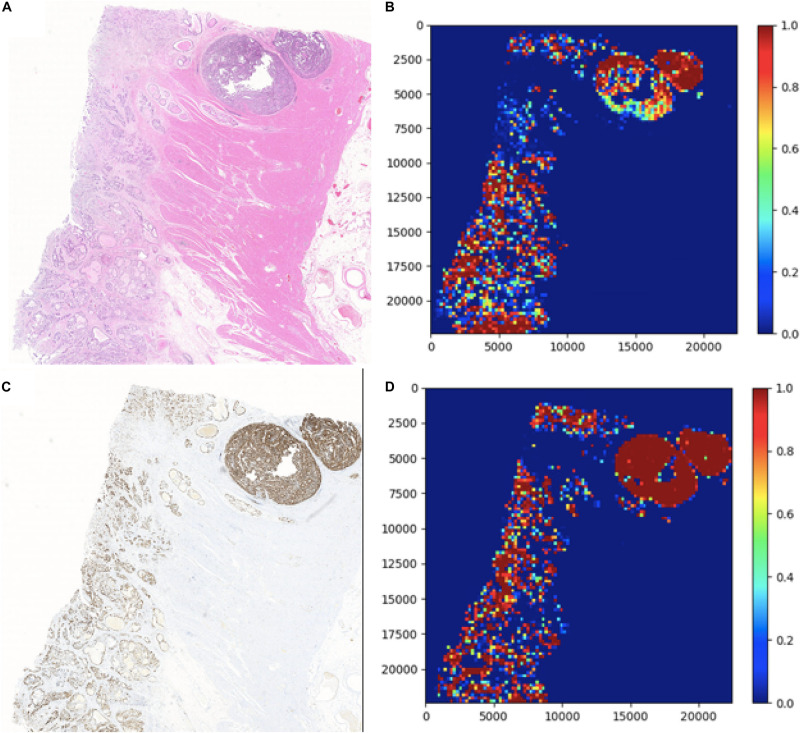
The image-level visualization of classification results in HE and Ki-67 domain: **(A)** is an HE pathology microscopy image, **(B)** is its heatmap, **(C)** is a Ki-67 pathology microscopy image, and **(D)** is its heatmap. Red is for tumor patches, and blue is for normal patches.

**FIGURE 8:**
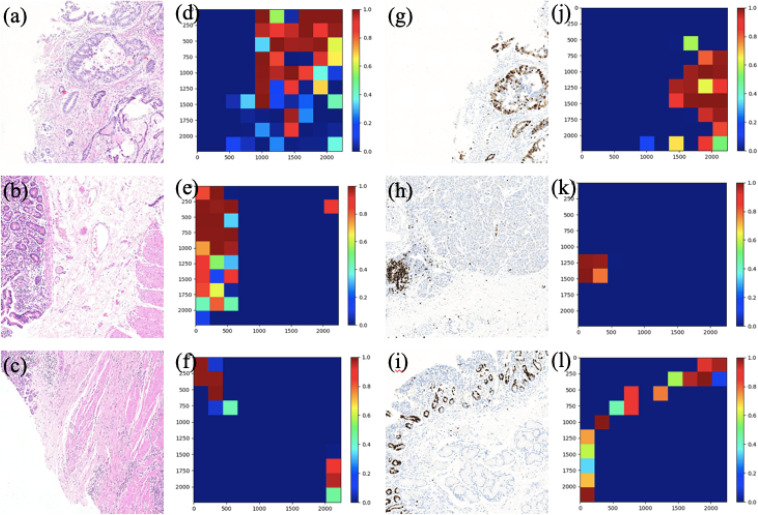
The patch-level visualization of classification results in HE and Ki-67 domain. Here we list six cases in three rows, three HE cases (left), and three Ki-67 cases (right): **(a–c)** are the HE pathology patches; **(d–f)** are their heatmaps; **(g–i)** are the Ki-67 pathology patches; and **(j–l)** are their heatmaps. Red is for tumor patches, and blue is for normal patches.

In this section, two figures (Figures 7, 8) show the experimental results of the binary classifiers in the HE domain and the Ki-67 domain using the MIL algorithm. Figure 7 shows the classification results in the HE domain and the Ki-67 domain, and they are at the image level. The image-level results show the average classification performance. Figure 8 shows the classification results in the HE domain and the Ki-67 domain, and they are at the patch level. The patch-level results show the classification performance in several regions with different densities of positive cells. From the visualization results, we can infer that we have got two classifiers for the tumor patch or the normal patch classification in the HE domain and the Ki-67 domain based on unannotated data.

### Visualization of Image Generation

**FIGURE 9:**
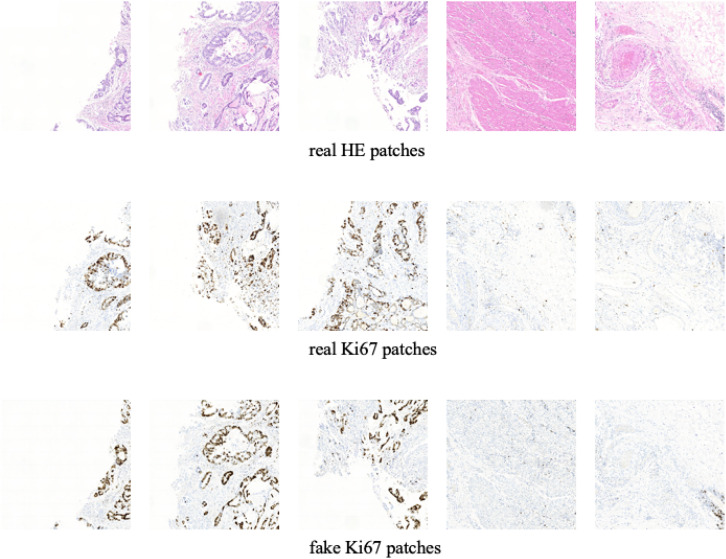
The patch-level visualization of image generation results. Here we list five cases in five columns. At each column, the top image is the HE pathology patch, the middle image is the corresponding Ki-67 pathology patch, and the bottom one is the synthetic Ki-67 pathology patch generated with our proposed method.

**FIGURE 10:**
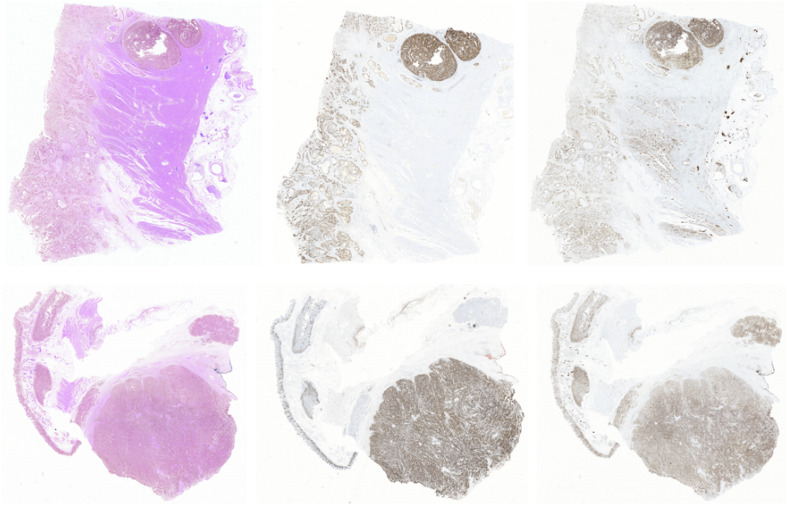
The image-level visualization of image generation results. Here we show two different cases in two rows. In each row, the left image is the HE pathology microscopy image, the middle image is the corresponding Ki-67 pathology microscopy image, and the right one is the synthetic Ki-67 pathology microscopy image generated with our proposed method.

In this section, we show the experimental results of our proposed Ki-67 pathology microscopy images generation method from HE pathology microscopy images, including patch-level visualization and image-level visualization. Figures 9, 10 show the patch-level visualization and the image-level visualization of our experimental results. Figure 9 shows five cases of our experimental results, in five columns. In each column, the top image is the HE patch, the middle image is the corresponding Ki-67 patch and the bottom image is the synthetic Ki-67 patch generated by our proposed method. Our proposed method is able to generate synthetic Ki-67 patches of high quality. By comparison with real Ki-67 patches, we can find that normal cells and tumor cells in HE patches can be transformed into normal cells and tumor cells in synthetic Ki-67 patches correctly. Figure 8 shows our experimental results at image level. In Figure 10, we show two different cases, in two rows. In each row, the left image is the original HE pathology microscopy image, the middle image is the corresponding Ki-67 pathology microscopy image, and the right image is the synthetic Ki-67 pathology microscopy image generated with our proposed method. We can easily find that our proposed method can work well in regions including background or a high rate of positive cells or a medium rate of positive cells. However, this result also shows that it cannot work well in regions with a low rate of positive cells. Clinically, when we examine Ki-67 pathology microscopy images, the color, dark or light, of positive cells is not important because the doctors are asked to count positive cells to make a diagnosis. Quantification results taking this clinical usage into consideration will be listed by illustrating the ratio of positive cells to all cells.

### Quantification Results

In this section, the ratio of positive cells to all cells will be set as the evaluating metric for our proposed method because of the clinical usage of Ki-67 pathology microscopy images. Each test HE pathology microscopy image will be fed into the well-trained generator to generate a synthetic Ki-67 pathology microscopy image. For quantification results, we separately count the ratio above from real Ki-67 pathology microscopy image and its corresponding synthetic Ki-67 pathology microscopy image, and the results are listed in Tables 3, 4.

**TABLE 3:** Quantification results 1 of our proposed method.

	**Training dataset**		**Evaluation dataset**	
	**Real Ki-67**	**Synthetic Ki-67**	**Real Ki-67**	**Synthetic Ki-67**
Ratio	0.3744	0.3850	0.3046	0.2771

*Ratio, ratio of the number of positive cells to the number of all cells. The results above show the potential value of our proposed method in clinical practice.*

**TABLE 4:** Quantification results 2 of our proposed method.

	**Example 1**		**Example 2**	
	**Real Ki-67**	**Synthetic Ki-67**	**Real Ki-67**	**Synthetic Ki-67**
Ratio	0.2570	0.2518	0.3522	0.3025

*Ratio, ratio of the number of positive cells to the number of all cells. The results above show the potential value of our proposed method in clinical practice.*

### Ablation Study

**FIGURE 11:**
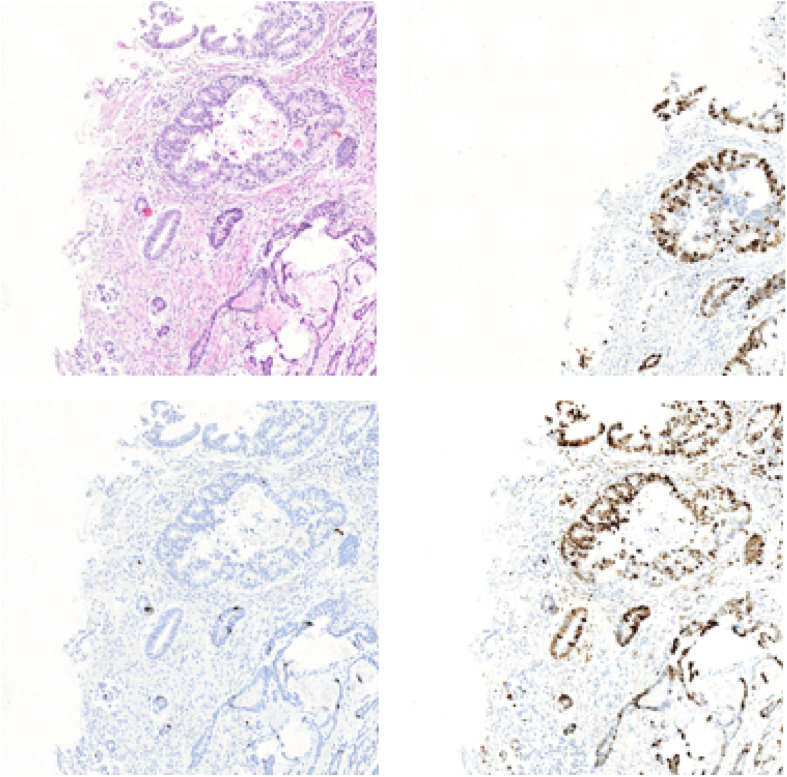
The representation of image generation results using raw CycleGAN and our proposed method. The top left image is the HE pathology patch, and the top right one is its corresponding Ki-67 pathology patch. The bottom left image is the result generated with only CycleGAN and the bottom right one is the result generated with our proposed method.

In this section, we will list the experimental results of Ki-67 pathology microscopy images generation from HE pathology microscopy images using only CycleGAN and our method. Figure 11 shows the visualization of the experimental results from only CycleGAN and our method at patch level. The top left one is the real HE patch, the top right one is the corresponding Ki-67 patch, the bottom left one is the synthetic Ki-67 patch generated only with CycleGAN, and the bottom right one is the synthetic Ki-67 patch generated with our method. The comparison means that our proposed method is effective in Ki-67 pathology microscopy image generation from HE pathology microscopy images.

## Discussion

This is the first attempt to apply CycleGAN for synthetic Ki-67 pathology microscopy images generation with an unpaired dataset. Moreover, the MIL algorithm has been adopted to extract class-related information from an unannotated dataset. Importantly, the idea behind conditional GAN is used to force the class-related feature vectors to be aligned during the adversarial training process. With all these methods, our proposed method is able to generate synthetic Ki-67 pathology microscopy images of high quality. Because our proposed method is working with an unannotated and unpaired dataset, our method can be applied to many other regions.

Although we propose a synthetic Ki-67 pathology microscopy images generation method and the performance is good enough, there exist many future works for us to address. The first one is the evaluation metric, which can evaluate its clinical usage. The second one is a larger dataset, which is essential to performance improvement. When addressing these problems, we believe we can obtain a model that can be used in clinical practice.

## Data Availability Statement

The raw data supporting the conclusions of this article will be made available by the authors, without undue reservation, to any qualified researcher.

## Author Contributions

ZX designed and conducted the experiments, analyzed the data, and drafted the manuscript. YH, XL, XZ, and YC guided the experiments and manuscript modification. All authors read and approved the final manuscript.

## Conflict of Interest

The authors declare that the research was conducted in the absence of any commercial or financial relationships that could be construed as a potential conflict of interest.

## References

[B1] Campanella G. Hanna M. G. Geneslaw L. Miraflor A. P. Silva V. W. Busam K. J. (2019). Clinical-grade computational pathology using weakly supervised deep learning on whole slide images. *Nat. Med.* 25 1301–1309. 10.1038/s41591-019-0508-1 31308507 PMC7418463

[B2] Frid-Adar M. Diamant I. Klang E. Amitai M. Goldberger J. Greenspan H. (2018a). GAN-based synthetic medical image augmentation for increased CNN performance in liver lesion classification. *Neurocomputing* 321 321–331. 10.1016/j.neucom.2018.09.013

[B3] Frid-Adar M. Klang E. Amitai M. Goldberger J. Greenspan H. (2018b). “Synthetic data augmentation using GAN for improved liver lesion classification,” in *2018 IEEE 15th International Symposium on Biomedical Imaging (ISBI 2018)*, 289–293. 10.1109/ISBI.2018.8363576

[B4] Goodfellow I. J. Pouget-Abadie J. Mirza M. Xu B. Warde-Farley D. Ozair S. (2014). Generative adversarial networks. *Adv Neural Inf. Process. Syst.* 3, 2672–2680.

[B5] Han C. Hayashi H. Rundo L. Araki R. Shimoda W. Muramatsu S. (2018). “GAN-based synthetic brain MR image generation,” in *International Symposium on Biomedical Imaging*, Washington, DC 10.1109/ISBI.2018.8363678

[B6] He K. Zhang X. Ren S. Sun J. (2016). “Deep residual learning for image recognition,” in *Proceedings of the IEEE Conference on Computer Vision and Pattern Recognition*, Las Vegas, NV, 770–778. 10.1109/CVPR.2016.90

[B7] Hoffman J. Tzeng E. Park T. Zhu J. Y. Isola P. Saenko K. (2017). “Cycada: cycle-consistent adversarial domain adaptation,” in *Proceedings of the 35th International Conference on Machine Learning, PMLR* 80, 1989–1998.

[B8] Kazeminia S. Baur C. Kuijper A. Ginneken B. V. Mukhopadhyay A. (2020). GANs for medical image analysis. *Artif. Intell. Medicine* 109:101938 10.1016/j.artmed.2020.101938 34756215

[B9] Li Y. Ping W. (2018). Cancer metastasis detection with neural conditional random field. Medical Imaging with Deep Learning. [Preprint]. arXiv:1806.07064.

[B10] Liu Y. Gadepalli K. Norouzi M. Dahl G. E. Stumpe M. C. (2017). “Detecting cancer metastases on gigapixel pathology images,” in *International Conference on Medical Image Computing and Computer Assisted Intervention*. [Preprint]. arXiv:1703.02442.

[B11] Mahmood F. Chen R. J. Durr N. J. (2018). Unsupervised reverse domain adaptation for synthetic medical images via adversarial training. *IEEE Trans. Med. Imaging* 37 2572–2581. 10.1109/tmi.2018.2842767 29993538

[B12] Mirza M. Osindero S. (2014). Conditional generative adversarial nets. [Preprint]. arXiv:1411.1784.

[B13] Nguyen T. D. Le T. Vu H. Phung D. (2017). “Dual discriminator generative adversarial nets. NIPS”, in *Proceedings of the 31st International Conference on Neural Information Processing Systems*, United States, 2667–2677.

[B14] Nie D. Trullo R. Lian J. Petitjean C. Ruan S. Wang Q. (2017). “Medical image synthesis with context-aware generative adversarial networks,” in *Medical Image Computing and Computer Assisted Intervention*, Quebec City 10.1007/978-3-319-66179-7_48 PMC6044459 30009283

[B15] Shin H. C. Tenenholtz N. A. Rogers J. K. Schwarz C. G. Senjem M. L. Gunter J. L. (2018). *Medical Image Synthesis for Data Augmentation and Anonymization Using Generative Adversarial Networks. Simulation and Synthesis in Medical Imaging. SASHIMI 2018. Lecture Notes in Computer Science*, New York: Springer International Publishing, 11037, 1–11. 10.1007/978-3-030-00536-8_1

[B16] Song C. He K. Wang L. Hopcroft J. E. (2019). “Improving the generalization of adversarial training with domain adaptation,” in *International Conference on Learning Representations*, Arizona: ICLR.

[B17] Wang D. Khosla A. Gargeya R. Irshad H. Beck A. H. (2016). Deep learning for identifying metastatic breast cancer. [Preprint]. arXiv:1606.05718.

[B18] Xu G. Song Z. Sun Z. Ku C. Yang Z. Liu C. (2019). “CAMEL: a weakly supervised learning framework for histopathology image segmentation,” in *International Conference on Computer Vision*, Piscataway, NJ: IEEE, 10682–10691. 10.1109/ICCV.2019.01078

[B19] Xu Z. Moro C. F. Bozóky B. Zhang Q (2019). Gan-based virtual re-staining: a promising solution for whole slide image analysis. *arXiv*:1901.04059.

[B20] Zhu J. Park T. Isola P. Efros A. A. (2017). “Unpaired image-to-image translation using cycle-consistent adversarial networks,” in *International Conference on Computer Vision*, Venice 10.1109/ICCV.2017.244

